# Prognostic significance of XB130 expression in surgically resected pancreatic ductal adenocarcinoma

**DOI:** 10.1186/1477-7819-12-49

**Published:** 2014-03-01

**Authors:** Jianli Zhang, Xiuli Jiang, Jian Zhang

**Affiliations:** 1 Department of General Surgery, The Affiliated Hospital of Qingdao University, 59 Haier Road, Qingdao 266000, Shandong Province, China; 2 Department of Nutrition, The Affiliated Hospital of Qingdao University, 59 Haier Road, Qingdao 266000, Shandong Province, China

**Keywords:** Pancreatic ductal adenocarcinoma, Immunohistochemistry, Prognosis, XB130

## Abstract

**Background:**

XB130 is a newly discovered adaptor protein for intracellular signal transduction; it is involved in gene regulation, cell proliferation, cell survival, cell migration, and tumorigenesis. However, its expression and role in pancreatic ductal adenocarcinoma (PDAC) have not been investigated. The present study was designed to clarify the prognostic significance of XB130 expression in PDAC.

**Methods:**

A total of 76 consecutive patients with surgically resected PDAC were retrospectively reviewed. XB130 expression was detected by immunohistochemical analysis on the paraffin-embedded tumour sections. Correlation between the expression of XB130 and clinicopathological parameters was analyzed.

**Results:**

XB130 expression was significantly upregulated in PDAC(56.5%, 43/76) compared to normal pancreas (0%, 0/15; *P* < 0.05). Increased XB130 expression was correlated with lymph node metastasis (*P* = 0.017), distant metastasis (*P* = 0.0024), high tumour-node-metastasis (TNM) stage (*P* =0.001), and high tumour grade (*P* = 0.013). The survival of 43 patients with high XB130 expression was significantly worse than that of the 33 patients with low XB130 expression (*P* = 0.001). Univariate analysis showed that high XB130 expression (*P* = 0.0045), tumour size (*P* = 0.024), distant metastasis (*P* = 0.003), TNM stage (*P* = 0.002) and lymphatic metastasis (*P* = 0.016) were independent prognostic factors of postoperative survival. Multivariate analysis using the Cox proportional hazards model showed that high XB130 expression and distant metastasis (*P* = 0.0239) were significant independent risk factors.

**Conclusions:**

XB130 was overexpressed in the PDAC. XB130 is a promising pathological marker for the prediction of outcome in patients with PDAC.

## Background

Pancreatic ductal adenocarcinoma (PDAC) is one of the most devastating human malignancies. Surgical resection remains the only potentially curative therapeutic option. At the time of initial diagnosis, only a minority of patients with PDAC are at a disease stage that can still potentially be cured by resection [1]. Even if a potentially curative resection can be performed, the 5-year overall survival is low at 10 to 25% [1-3]. Because of the lack of methods for the early diagnosis and limited knowledge on the biological features of PDAC, the majority of patients are not diagnosed properly until the advanced stage [4]. Prognostic factors for PDAC have been well studied, and include gender, age, size and location of the tumour, stage, lymph node metastasis, tumour grade, and serum carbohydrate antigen 19-9 level [1,2,5,6]. However, none of these established clinical markers have correlated with outcome and therapeutic response in patients with PDAC [7].

XB130 is a newly discovered adaptor protein for intracellular signal transduction; it is involved in gene regulation, cell proliferation, cell survival, cell migration, and tumorigenesis [8]. XB130 is strongly expressed in the spleen and thyroid of humans, while it shows weak expression in the kidney, brain, lung, and pancreas [9]. XB130 has been detected in follicular and papillary thyroid carcinoma, human lung carcinoma cell lines [10], human oesophageal squamous cell carcinoma [11], hepatocellular carcinoma [12] as well as in gastric cancer [13]. In gastric cancer, reduced XB130 protein expression is a prognostic biomarker for shorter survival and a higher recurrence rate in patients with gastric cancer, as well as for the response to chemotherapy [13]. In oesophageal squamous cell carcinoma (ESCC), the expression of XB130 in ESCC cells may affect cell cycle progression and impact prognosis of patients with ESCC [11].

This study examines the expression of XB130 in 76 resected PDAC patients by immunohistochemistry and investigates the correlation between XB130 expression and prognosis.

## Methods

### Patients

We analysed 76 consecutive patients with PDAC who underwent surgical resection in the Department of General Surgery at the affiliated hospital of Qingdao University between March 2003 and February 2009. Patients were excluded from the study if they had a previous history of another malignancy, or had received chemotherapy or radiotherapy before surgical resection, or had undergone palliative resection. Surgical procedures were as follows: 43 patients were treated by conventional pancreatoduodenectomy, 18 were treated by pylorus preserving pancreatoduodenectomy, 8 were treated by distal pancreatectomy, and 4 were treated by total pancreatectomy. The age of the patients ranged from 23 to 72 years, and the median age was 64 years. All surgical specimens were reviewed and classified according to the World Health Organization classification by an experienced pathologist who was unaware of clinical or imaging findings. Pathological tumour-node-metastasis (TNM) stages were established using the International System for Staging Pancreatic ductal adenocarcinoma adopted by the American Joint Committee on Cancer and the Union Internationale Centre le Cancer [13]. Of the total patients, 12, 39, 19 and 6 had stage I, II, III and IV tumours, respectively. Postoperative adjuvant chemotherapy with gemcitabine, 5-fluorouracil(5-Fu) and oral administration of tegafur (a fluorouracil derivative drug) were administered to 21, 10 and 1 patients, respectively. The day of surgery was considered the starting day for measuring postoperative survival.

A control group consisted of seven patients with benign pancreatic lesions (one patient with intraductal papillary-mucinous adenoma, two patients with solid-pseudopapillary tumour, one patient with lymphoplasmacytic sclerosing pancreatitis, one patient with chronic sclerosing pancreatitis, one patient with inflammatory pseudotumour and one patient with pancreatic endocrine cell tumour), five cases of pancreatic lesions from traumatic injury of the pancreas and an organ donor program from three previously healthy individuals (two female, one male; median age of 31 years, with a range of 18 to 56 years) when there was no suitable recipient. The study protocol was approved by the institution of the affiliated hospital of Qingdao University.

### Immunohistochemistry

Rabbit anti-XB130 Ab (1:100; (Abnova, Shanghai, China) was used as primary antibody. Immunohistochemical staining was performed with an immunoperoxidase method using the ABC complex as the manufacturer’s recommended protocol. Briefly, each section was dewaxed with xylene. Endogenous peroxidase was blocked by incubating the sections in 0.3% hydrogen peroxidase in absolute methanol at room temperature for 30 minutes. After hydration in decreasing concentrations of ethanol in water, the sections were washed in 0.01 M PBS, pH 7.4. Antigen retrieval was achieved by waterbath pretreatment at 80°C for 20 minutes in 0.01 M citrate buffer (pH 6.0). The sections were washed twice with PBS and 2% horse or goat serum in PBS was applied for 30 minutes at room temperature to prevent non-specific staining. The sections were then incubated with dilutions of the anti-XB130 in PBS with 1% bovine serum albumin for 16 hours at 4°C. The sections were washed three times with PBS, incubated with the biotinylated secondary antibodies, and then washed three times with PBS. All sections then received ABC complex for 30 minutes. After washing with PBS three times, the sections were finally reacted with diaminobenzidine substrate for 10 minutes for visualisation, rinsed with tap water, counterstained with haematoxylin, and mounted. Reaction products were not present when non-immune serum or PBS was used instead of the primary antibodies.

Immunohistochemistry results were evaluated by scanning each slide under low power magnification (×10) to identify regions containing positive immunoreactivity. Immunostaining was further evaluated at high power magnification (×200). The percentages of positively stained cells ≤25% were considered to be low expression, and >25% were considered to be high expression. XB130 immunostaining was evaluated independently by two individuals blinded to the clinical parameters.

### Statistical analysis

The results are presented as mean ± SD. Statistical analysis was performed using the Student’s *t* test, the chi^2^ test, and the Mann-Whitney U test where appropriate. Univariate and multivariate survival analyses were performed using the Cox proportional hazards regression model. Furthermore, backward stepwise multivariate analysis was used to find independent prognostic factors. A value of *P* < 0.05 was considered significant. Statistical analysis of the data was performed using SPSS software version 10.0 (SPSS Inc., Chicago, IL, USA).

## Results

### Immunohistochemical analysis

The immunohistochemical analysis of XB130 was performed on the 76 primary lesions with PDAC and seven resected lesions with benign pancreatic diseases, five cases of pancreatic lesions from traumatic injury of the pancreas and an organ donor program from three previously healthy individuals. XB130 immunostaining was detected in carcinoma cells in the tumour tissues. It was localised predominantly on the cytoplasm. In the 76 patients with PDAC, high XB130 expression was recognized in 56.5% (43/76) of cases, which was significantly high than the XB130 expression in the normal pancreas (0%, 0/15) (*P* < 0.05).

### Prognostic value of XB130 expression and clinicopathologic variables

We investigated the relationship between XB130 protein expression and various clinicopathological features in PDAC (Table 1). No correlation could be observed between tumor XB130 expression and age, gender, tumour size, histologic differentiation, lymphatic invasion, vascular invasion, perineural invasion and chemotherapy status. In contrast, increased XB130 expression was correlated with lymph node metastasis (*P* = 0.017), distant metastasis (*P* = 0.0024), high TNM stage (*P* = 0.001), and high tumour grade (*P* = 0.013).

**Table 1:** Relation between XB130 expression and clinicopathologic variables in pancreatic cancer patients

		**XB130 expression**		
**Variables**	**Number**	**High (n)**	**Low (n)**	***P *** **value**
Gender				ns
Male	48	31	17	
Female	28	12	16	
Age (years)				ns
<60	45	29	16	
≥60	31	14	17	
TNM stage				0.001
I	12	3	9	
II	39	18	21	
III	19	16	3	
IV	6	6	0	
Tumour size				ns
≤2 cm	50	32	18	
>2 cm	26	11	15	
T classification				0.013
T1/T2	15	4	11	
T3/T4	61	39	22	
N classification				0.017
N0	25	8	17	
N1	51	35	16	
Histologic differentiation				ns
Well (G1)	30	18	12	
Moderate (G2)	28	15	13	
Poor (G3)	18	10	8	
Distant metastasis				0.0024
M0	60	29	31	
M1	16	14	2	
Lymphatic invasion				ns
Yes	14	8	6	
No	59	35	24	
Vascular invasion				ns
Yes	18	10	8	
No	58	33	25	
Perineural invasion				ns
Yes	8	3	5	
No	68	40	28	
Chemotherapy				ns
No	32	20	12	
Yes	44	23	21	

ns, Not significant; TNM, tumour-node-metastasis.

**Figure 1:**
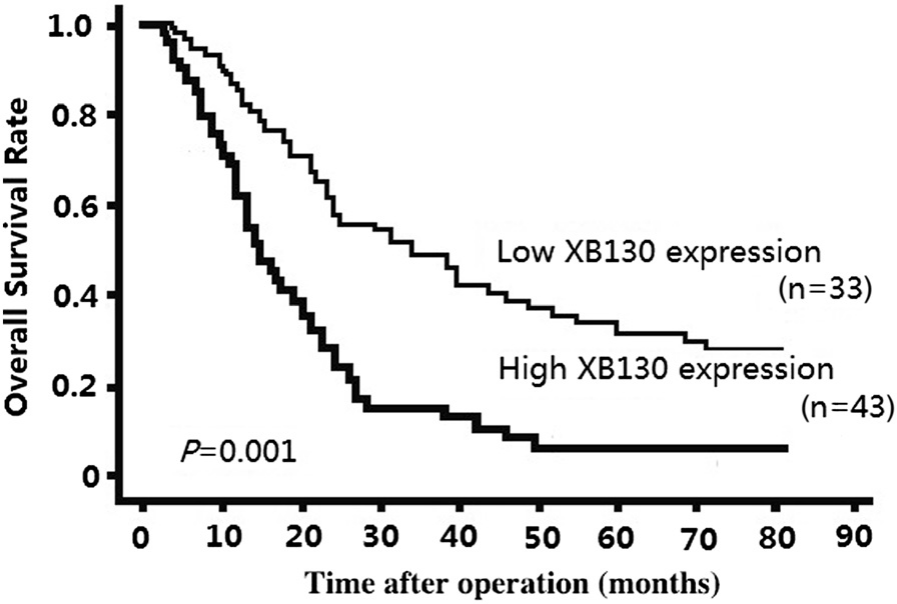
**Overall survival of 76 pancreatic ductal adenocarcinoma patients in relation to XB130 protein expression.** Kaplan-Meier survival curves for patients who had undergone surgical resection of pancreatic ductal adenocarcinoma, stratified according to the level of expression of XB30 in their tumours. Patients with low tumor XB130 protein expression had a significantly better prognosis than patients with high tumour XB130 protein expression (log-rank test, *P* = 0.001).

The survival curves of the patients, grouped according to the level of XB130 staining in their tumours, are shown in Figure 1. The high XB130 expression group had a significantly poorer prognosis than the low XB130 expression group (log-rank test, *P* = 0.001).

Univariate analysis showed that high XB130 expression (*P* = 0.0045), tumour size (*P* = 0.024), distant metastasis (*P* = 0.003), TNM stage (*P* = 0.002) and lymphatic metastasis (*P* = 0.016) were independent prognostic factors of postoperative survival. Multivariate analysis using the Cox proportional hazards model showed that high XB130 expression (*P* = 0.0043) and distant metastasis (*P* = 0.0239) were significant independent risk factors (Table 2).

**Table 2:** Prognostic factors in Cox’s proportional hazards model

	**Univariate**			**Multivariate**		
Gender	HR	95% CI	*P*	HR	95% CI	*P*
Male/female	1.340	0.847-1.952	0.438			
Age (years)						
<60/≥60	0.982	0.674-1.283	0.572			
TNM stage						
I-II/III- IV	2.542	1.734-5.863	0.002	1.84	1.546-2.874	0.056
Tumour size						
≤2 cm/>2 cm	1.845	1.38-4.962	0.024	1.632	1.145-2.247	0.134
T classification						
T1-T2/T3-T4	1.472	0.863-2.168	0.386			
N classification						
N0/N1	2.389	1.473-3.974	0.016	1.357	0.964-2.832	0.106
Histologic differentiation						
G1/G2-G3						
Distant metastasis						
M0/M1	2.167	1.168-5.94	0.003	2.21	1.145-5.263	0.0239
Lymphatic invasion						
Yes/no	1.149	0.793-2.865	0.067			
Vascular invasion						
Yes/no	1.354	0.872-2.004	0.138			
Perineural invasion						
Yes/no	1.457	0.641-2.359	0.275			
Chemotherapy						
Yes/no	1.580	0.794-2.356	0.192			
XB130 expression						
Low/high	2.396	1.273-3.944	0.0045	2.375	1.263-3.874	0.0043

HR, hazard ratio; TNM, tumour-node-metastasis.

## Discussion and conclusions

To establish proper therapeutic modalities for PDAC, an accurate assessment of the factors affecting tumouTNM staging system, which is defined by tumour size, tumour progression, lymph node involvement, and distant metastasis [14] is useful for PDAC classification, the outcome is poor for patients even in the low-stage (I and II) groups [15]. Therefore, the prognostic use of several molecular markers for PDAC classification have been investigated [16], although none proved useful for predicting patient prognosis [7,17]. We undertook the present study to determine whether XB130 expression is the a valid biological indicator of the aggressiveness of PDAC.

Recent studies have shown that high XB130 expression is significantly associated with cell proliferation, angiogenesis and poor outcome in patients with various human neoplasms [8,10-13]. However, little is known regarding the clinical significance of XB130 expression in human cancer, such as PDAC. In the present study, XB130 was highly expressed in PDAC cells compared with normal pancreatic cells, and the high expression of XB130 protein within PDAC cells closely correlated with high TNM stage, distant metastasis, high T and N classification and dismal postoperative survival. These results suggest that over-expression of XB130 might enhance cell motility and invasiveness. It is also clearly demonstrated that the expression of XB130 was a significant independent factor for predicting poor survival outcome in patients with surgically resected PDAC.

A previous review has summarised the immunohistochemical biomarkers with prognostic significance in patients with PDAC and concluded that none of the molecular markers can be recommended for routine clinical use [18]. Therefore, whether the presence of these molecular markers has any prognostic implications remains unclear. The results of our study identified the XB130 as an independent prognostic factor for predicting poor outcome. Although a recent retrospective study has demonstrated that patients with adjuvant therapy have more adverse prognostic factors than those without adjuvant therapy [19], XB130 was associated with prognostic significance regardless of adjuvant therapy.

In conclusion, high expression of XB130 (measured by immunohistochemical staining) can serve as an independent prognostic marker to predict poor outcome after surgical resection and may be an important clinical marker of therapy for PDAC. Inhibition of XB130 function may arrest tumour growth, and XB130 represents an attractive target for adjuvant therapy in the future.

## Abbreviations

ESCC: oesophageal squamous cell carcinoma; PBS: phosphate buffered saline; PDAC: pancreatic ductal adenocarcinoma; TNM: tumour-node-metastasis.

## Competing interests

The authors declare that they have no competing interests.

## Authors’ contributions

JliZ and XJ performed the majority of experiments; JZ collected all the human materials; JliZ and XJ designed the study and wrote the manuscript; JliZ was involved in editing the manuscript. All authors read and approved the final manuscript.

## References

[B1] Geer RJ Brennan MF Prognostic indicators for survival after resection of pancreatic adenocarcinoma Am J Surg 1993 165 68 72 10.1016/S0002-9610(05)80406-4 8380315

[B2] Cameron JL Riall TS Coleman J Belcher KA One thousand consecutive pancreaticoduodenectomies Ann Surg 2006 244 10 15 10.1097/01.sla.0000217673.04165.ea 16794383 PMC1570590

[B3] Kleeff J Michalski C Friess H Buchler MW Pancreatic ductal adenocarcinoma: from bench to 5-year survival Pancreas 2006 33 111 118 10.1097/01.mpa.0000229010.62538.f2 16868475

[B4] Li C Heidt DG Dalerba P Burant CF Zhang L Adsay V Wicha M Clarke MF Simeone DM Identification of pancreatic ductal adenocarcinoma stem cells Cancer Res 2007 67 1030 1037 10.1158/0008-5472.CAN-06-2030 17283135

[B5] Ueda M Endo I Nakashima M Minami Y Takeda K Matsuo K Prognostic factors after resection of pancreatic ductal adenocarcinoma World J Surg 2009 33 104 110 10.1007/s00268-008-9807-2 19011933

[B6] Lee KJ Yi SW Chung MJ Park SW Song SY Chung JB Serum CA 19-9 and CEA levels as a prognostic factor in pancreatic adenocarcinoma Yonsei Med J 2013 54 643 649 10.3349/ymj.2013.54.3.643 23549809 PMC3635646

[B7] Hilgers W Kern SE Molecular genetic basis of pancreatic adenocarcinoma Genes Chromosomes Cancer 1999 26 1 12 10.1002/(SICI)1098-2264(199909)26:1<1::AID-GCC1>3.0.CO;2-X 10440999

[B8] Shiozaki A Liu M Roles of XB130, a novel adaptor protein, in cancer J Clin Bioinforma 2011 1 10 10.1186/2043-9113-1-10 21884627 PMC3164603

[B9] Xu J Bai XH Lodyga M Han B Xiao H XB130, a novel adaptor protein for signal transduction J Biol Chem 2007 282 16401 16412 10.1074/jbc.M701684200 17412687

[B10] Shiozaki A Lodyga M Bai XH Nadesalingam J Oyaizu T XB130, a novel adaptor protein, promotes thyroid tumor growth Am J Pathol 2011 178 391 401 10.1016/j.ajpath.2010.11.024 21224076 PMC3070596

[B11] Shiozaki A Kosuga T Ichikawa D Komatsu S Fujiwara H Okamoto K Iitaka D Nakashima S Shimizu H Ishimoto T Kitagawa M Nakou Y Kishimoto M Liu M Otsuji E XB130 as an independent prognostic factor in human esophageal squamous cell carcinoma Ann Surg Oncol 2013 20 3140 3150 10.1245/s10434-012-2474-4 22805860

[B12] Zuo Q Huang H Shi M Zhang F Sun J Bin J Liao Y Liao W Multivariate analysis of several molecular markers and clinicopathological features in postoperative prognosis of hepatocellular carcinoma Anat Rec (Hoboken) 2012 295 423 431 10.1002/ar.21531 22190283

[B13] Shi M Huang W Lin L Zheng D Zuo Q Wang L Wang N Wu Y Liao Y Liao W Silencing of XB130 is associated with both the prognosis and chemosensitivity of gastric cancer PLoS One 2012 7 e41660 10.1371/journal.pone.0041660 22927913 PMC3426513

[B14] Sobin LH Gospodarowicz MK Ch W International Union Against Cancer (UICC) TNM Classification of Malignant Tumours 2009 7 Oxford, UK: Wiley-Blackwell

[B15] Sobin LH Wittekind CH TNM Classification of Malignant Tumours 2002 6 New York: John Wiley & Sons 93 96

[B16] Mukaiya M Hirata K Satoh T Lack of survival benefit of extended lymph node dissection for ductal adenocarcinoma of the head of the pancreas: retrospective multi-institutional analysis in Japan World J Surg 1998 22 248 252 10.1007/s002689900378 9494416

[B17] Ghaneh P Kawesha A Evans JD Neoptolemos JP Molecular prognostic markers in pancreatic ductal adenocarcinoma J Hepatobiliary Pancreat Surg 2002 9 1 11 10.1007/s005340200000 12021893

[B18] Ansari D Rosendahl A Elebro J Andersson R Systematic review of immunohistochemical biomarkers to identify prognostic subgroups of patients with pancreatic ductal adenocarcinoma Br J Surg 2011 98 1041 1055 10.1002/bjs.7574 21644238

[B19] Corsini MM Miller RC Haddock MG Donohue JH Farnel MB Nagorney DM Jatoi A McWilliams RR Kim GP Bhatia S Iott MJ Gunderson LL Adjuvant radiotherapy and chemotherapy for pancreatic carcinoma: the Mayo Clinic Experience (1975-2005) J Clin Oncol 2008 26 3511 3516 10.1200/JCO.2007.15.8782 18640932

